# Step by step: promoting daily walking through a mHealth intervention

**DOI:** 10.3389/fpsyg.2026.1795308

**Published:** 2026-03-25

**Authors:** Roberta Adorni, Maria Elide Vanutelli, Gabriele Damaschi, Marco D’Addario, Patrizia Steca

**Affiliations:** Department of Psychology, University of Milan-Bicocca, Milan, Italy

**Keywords:** BCTs, behavioral goal setting, HAPA, mHealth, rewards, self-monitoring, walking behavior

## Abstract

**Background:**

Mobile health (mHealth) interventions are increasingly used to promote individual well-being. Mobile applications provide accessible, personalized tools for monitoring and improving health, thereby fostering the adoption of positive behaviors. Nonetheless, further evidence is needed to support the effectiveness of mHealth programs. The present study aims to evaluate the effectiveness of a protocol designed to increase daily step counts, grounded in the Health Action Process Approach (HAPA).

**Methods:**

The protocol was delivered via the MyPocketHealth app, developed by the research team, and incorporated different communication strategies. Participants were involved in setting a goal and choosing between a personalized or a standardized goal (7,000 daily steps), which could be reviewed at any time. Moreover, they received either interactive (HAPA-I: group 1) or non-interactive (HAPA-NI: group 2) daily notifications promoting the target behavior through HAPA constructs for 15 days, while also monitoring their daily steps (self-monitoring). A third group engaged solely in self-monitoring without receiving any communication (no-communication group; No comm). Goal attainment was reinforced through badge awards (rewards). A sample of 210 healthy, physically inactive participants aged 22 to 55 underwent the intervention.

**Results:**

Significantly higher dropout rates were recorded in the HAPA-NI group compared to the other experimental conditions. However, participants in the HAPA-NI and HAPA-I groups showed gradual increases in daily step counts relative to baseline throughout the intervention period. Conversely, participants in the No-communication group showed a marked increase in daily step counts relative to baseline during the initial phase of the intervention, which progressively declined over time.

**Conclusion:**

These findings suggest that theory-driven notifications effectively promote sustained engagement in achieving behavioral goals, and that it is crucial to optimize the balance between notification frequency and interactivity when designing mHealth interventions to improve user engagement and ensure long-term effectiveness.

## 1 Introduction

Regular physical activity is a key strategy for maintaining health and improving well-being. On the other hand, physical inactivity is strongly associated with a higher risk of various adverse health outcomes, including non-communicable diseases (NCDs), which is a critically growing burden on healthcare systems (World Health Organization, 2025). The World Health Organization recommends at least 150 min of moderate or vigorous physical activity per week, along with reducing sedentary time (World Health Organization, 2024b). A wholesome habit is covering 7,000 steps a day, equivalent to about an hour of walking (Tudor-Locke and Bassett, 2004; Garber et al., 2024; Paluch et al., 2021). The worldwide picture is critical: in 2022, 31% of adults (1.8 billion people) did not meet the recommended levels of physical activity, with projections indicating a further increase to 35% by 2030 (World Health Organization, 2024a). Gender differences are also evident: 34% of women are inactive compared to 29% of men (World Health Organization, 2024a). These data underline the need for effective targeted interventions, in line with the Global Action Plan on Physical Activity 2018–2030 (World Health Organization, 2018).

To address this scenario, a promising approach consists of digital interventions. The literature has highlighted an increasing variety of interventions aimed at promoting physical activity and reducing sedentary behavior, ranging from web-based programs to wearable devices and mobile health applications (mHealth apps) (Davis et al., 2020; Singh et al., 2024). Among these, apps have emerged as an effective tool, with multiple studies demonstrating their ability to foster behavior change (Rodríguez-González et al., 2023; Wang et al., 2024).

In fact, mHealth apps are cost-effective, personalized, and feature-rich tools that provide visual feedback on health behaviors and support low-contact, remote, and tailored interventions (Wang et al., 2024). Numerous app-based interventions have been developed to promote physical activity, with their effectiveness often shaped by different study designs and underlying assumptions. Several authors emphasize the importance of building interventions upon a solid theoretical foundation (Wunsch et al., 2024). Additionally, app features may vary across studies, ranging from the inclusion of diverse behavior change techniques (BCTs) (Wunsch et al., 2024) to elements of gamification or personalization, which are considered key aspects for participants’ engagement and consequent adherence (Jakob et al., 2022). Furthermore, studies may incorporate additional methods and tools beyond the intervention app, such as questionnaires, interviews (Maes et al., 2024), educational materials (Pomkai et al., 2024), or messaging apps to receive feedback (Figueroa et al., 2022).

Despite increasing evidence that app-based interventions can successfully promote physical activity, the literature highlights the need to carefully design them with regard to context, duration, and target population (Rodríguez-González et al., 2023). It underscores practical issues such as inconsistencies in step counts between apps and wearables (Maes et al., 2024) and potential interference from other apps (Lugones-Sanchez et al., 2022). In addition, self-reported measures, though widely used, can show incongruences with objective data, likely due to desirability bias (Chukwuemeka et al., 2025). Moreover, studies on this topic are susceptible to self-selection bias, as participants who volunteer for physical activity research may already be more active and motivated (Cheung et al., 2025). Another central concern in these studies is maintaining participants’ engagement over time, which is crucial to minimizing attrition (Lugones-Sanchez et al., 2022; Pomkai et al., 2024). Achieving this over extended periods is particularly difficult and complicates the investigation of the long-term effectiveness of these interventions, which remains underexplored (Zhang et al., 2022). Methodologically, few studies have employed rigorously randomized controlled designs with blinding, and the impact of digital notification design, participant satisfaction, and app usability on intervention effectiveness is still unclear (Galavi et al., 2024). Ultimately, there is a need for interventions grounded in a precise theoretical framework that incorporates relevant psychological constructs (Michie et al., 2011; Wunsch et al., 2024), with particular attention to psychological outcomes relevant to behavioral change (Chukwuemeka et al., 2025).

Building on our previous study (Adorni et al., 2025), the present study implemented an *ad hoc* app (namely, “MyPocketHealth”) designed to promote physical activity. The intervention was grounded in the Health Action Process Approach (HAPA) (Schwarzer, 2008), a widely used behavioral change model that conceptualizes health behavior change as a staged process. In the motivational phase, individuals form the intention to change, influenced by action self-efficacy, risk perception, and outcome expectancies. In the subsequent volitional phase, behavior is adopted and maintained, supported by planning strategies, maintenance self-efficacy, and recovery self-efficacy.

Employing four BCTs (behavioral goal setting, prompt review of behavioral goals, prompt self-monitoring of behavior, and providing rewards contingent on successful behavior) according to the CALO-RE taxonomy (Michie et al., 2011), the intervention was structured into three experimental conditions differing in the type of digital communication provided.

The primary aim was to evaluate the effectiveness of the intervention in fostering a positive change in walking behavior. In addition, the study examined how variations in notification design influenced behavioral outcomes, participants’ engagement, and app usability, with the aim of elucidating the mechanisms through which the intervention promoted physical activity. The association between socio-demographic characteristics, external contextual factors (i.e., changes in daily routines), and walking behavior was also examined to identify meaningful patterns and generate insights relevant for future research and practical applications.

The novelty of the present study lies in the implementation of personalized feedback notifications systematically grounded in the Health Action Process Approach, which informed both their content and sequencing. Furthermore, the intervention aligns with the Ontology of Behavior Change Interventions model (Michie et al., 2018) by explicitly considering exposure to the intervention, the psychological mechanisms underpinning behavior change, and the contextual factors influencing walking outcomes. By integrating multiple clearly specified BCTs within a theoretically grounded digital framework, the study aims to offer a transparent, theory-driven, and replicable contribution to the literature.

Specifically, the study was designed to test the following hypotheses:

*H1*: The use of the app-based intervention would result in a significant improvement in walking behavior over time.

*H2*: The type of digital communication delivered during the intervention—HAPA-Interactive, HAPA-Non-Interactive, or no communication—would significantly influence participants’ average step count. In particular, notifications grounded in the Health Action Process Approach were expected to positively affect walking behavior, with the strongest impact anticipated in the interactive condition.

*H3*: Participants’ engagement with the intervention and perceived usability of the application would be satisfactory overall, with higher levels expected in the HAPA-Interactive condition compared to the other experimental groups.

## 2 Materials and methods

### 2.1 Participants

Participants were recruited through the Bilendi online platform.^1^ Bilendi S.r.l. is a panel provider offering innovative solutions for the collection and management of quantitative and qualitative research data, operating in Europe and the United States. It provided a financial incentive of 7 euros. Eligibility criteria for participation were assessed using an online recruitment questionnaire, administered to a large representation of the panel. They included the following: participants must be aged between 18 and 55, report being in good physical health, report being physically inactive, and own a smartphone (either iPhone or Android) along with a smartwatch for tracking steps. Individuals were excluded from participation if they reported taking at least 7,000 steps per day, engaged in continuous physical activity (as measured by the IPAQ, detailed in Section 2.3), or had a specific medical condition that would prevent them from voluntarily modifying their lifestyle without medical guidance. Health information was gathered through 10 specific questions covering symptoms such as cardiovascular/pulmonary health, orthopedic issues, and drug prescription. These questions were formulated by a physician who collaborated during the project’s initial phase.

A total of 290 panelists completed the first questionnaire (T0) and recorded at least 60% of their daily step entries. However, 80 panelists dropped out and did not complete the final questionnaire (T1). Therefore, the analyses included 210 panelists (Table 1). They were all from Italy; the majority of the sample consisted of women (69%), and they had a mean age of 41 (SD = 8.85, range 22–55). Almost half of the participants (43%) earned a high school degree. The majority of the sample was employed (81%).

**Table 1:** Sociodemographic characteristics of the final sample (*n* = 210).

Sociodemographic variables	
Variables	Values
Age (mean ± SD; range)	40.68 ± 8.85 (22–55)
Gender, *n* (%)	
Male	66 (31%)
Female	144 (69%)
Education, *n* (%)	
Middle school diploma	4 (2%)
High school diploma	91 (43%)
Bachelor’s degree	33 (16%)
Master’s degree	67 (32%)
Postgraduate training	15 (7%)
Occupation, *n* (%)	
Student	14 (7%)
Worker	170 (81%)
Unemployed	12 (6%)
Housekeeper	14 (7%)

The adequacy of the sample size was determined through power analysis, as outlined by Cohen (2013). G*Power Version 3.1.9.7 (Faul et al., 2007) was used, except for the linear mixed model (LMM), which was run in RStudio (version 2025.09.2 + 418, *Cucumberleaf Sunflower*). The required sample sizes for each analysis included in the study were calculated. Below, the sample size calculation for the analysis that required the largest sample was provided, specifically the Mann–Whitney *U* test. This test was used to identify potential differences between participants who completed the study and those who dropped out. For this test, the following parameters were used: effect size (*d*) = 0.5 (medium), *α* = 0.05, power = 0.90, and allocation ratio = 0.38. The calculated sample size was 224 individuals. Regarding the LMM, the *a priori* Monte Carlo simulation-based power analysis was conducted in R using the *simr* and *pacman* packages. The following parameters were set: small and medium effect sizes, α = 0.05, power = 0.90, ICC = 0.30, and 12 repeated measurements. Results indicated that a sample size greater than 65 participants (*N* > 65) was sufficient to achieve the target power.

Taking these factors into account, the study’s sample size was deemed sufficient to detect medium-sized effects.

The research was evaluated and approved by the local commission for minimal-risk studies of the Psychology Department of the University of Milan-Bicocca (protocol code: RM-2021-482).

This study represents an improved version of a previous protocol, pre-registered at ClinicalTrials.gov (Trial ID: NCT05620888) and published by Adorni et al. (2025). Two main modifications were introduced. First, the intervention duration was reduced to 15 consecutive days to enhance feasibility and adherence while preserving its core components. Second, the interactive HAPA notification system was revised from a baseline-driven model to a dynamic approach, with notifications generated based on participants’ daily responses during the intervention. All other methodological aspects remained consistent with the original protocol unless otherwise specified.

All participants were informed about the study purposes and procedures and voluntarily provided consent to participate. The present research was conducted in accordance with the principles outlined in the Declaration of Helsinki.

### 2.2 Procedure

**Figure 1:**
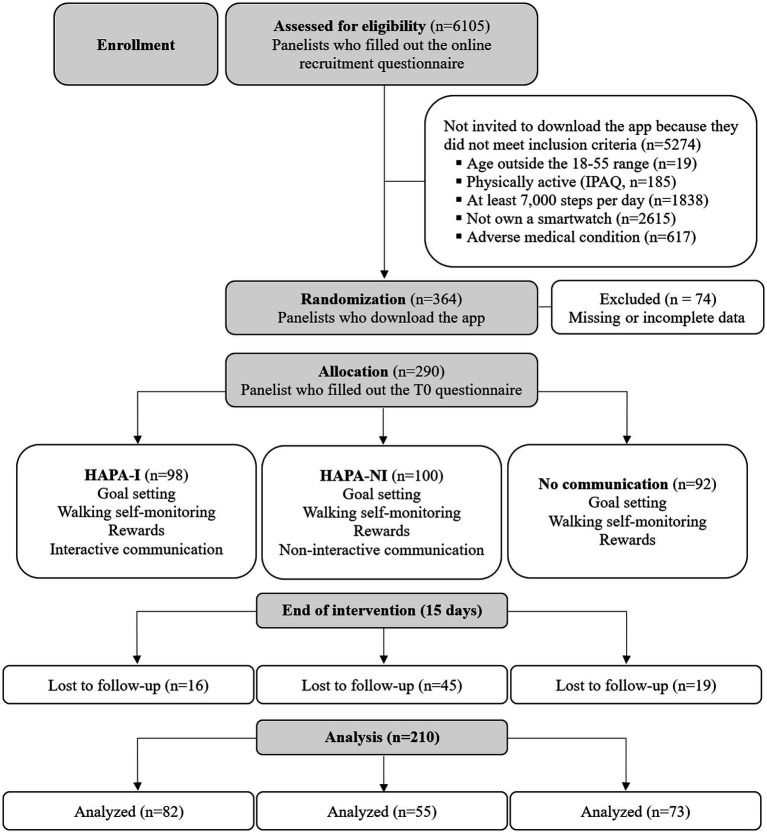
Flowchart of the study.

The study used a prospective, three-arm, parallel-group, randomized design, which included a baseline assessment followed by a 15-day trial period (Figure 1).

We developed a mobile application called *MyPocketHealth*, which encouraged users to participate in a physical activity program. Participants were tasked with achieving a daily goal, either by following the standard recommendation of 7,000 steps or setting a personalized goal. They were also required to self-monitor their progress by logging the number of steps taken each day in the app. To facilitate engagement, the app awarded a medal each day the user achieved the set goal. The application was developed by IMoobyte S.r.l., and its further development was managed by Archimedia S.r.l., both of which are software companies based in Italy.

Before enrolling in the study, all potential participants completed a brief recruitment questionnaire to determine eligibility. The questionnaire landing page explained the study’s purposes and procedures and obtained informed consent from the volunteers. A series of questions was then utilized to assess their eligibility, as outlined in Section 2.1.

Eligible volunteers were invited to download the app and join the study. The app automatically assigned each participant to one of three experimental conditions using a round-robin, single-blinded randomization method (1:1:1). At the conclusion of the 15-day intervention, participants received a notification from the app inviting them to complete an online questionnaire (T1), which assessed engagement with the intervention and usability of the application.

Participants in the “HAPA-I group” received two daily notifications, delivered at 10 a.m. and 4 p.m. Each notification contained a question related to a variable from the HAPA (as described in the Supplementary material). For example, one question asked, “Do you feel capable of reaching your step goal today? (Action Self-Efficacy).” Participants responded using a 5-point Likert scale, where 1 indicated “Not at all capable” and 5 signified “Very capable.” Those who scored low (1–3) received a motivational message tailored to their response. For example, “Do not be discouraged: with good planning and the right strategies, you can succeed without much effort!” In contrast, participants who scored high (4–5) received messages like, “Great! This attitude is a good starting point for reaching your goal!” The content of the notifications was crafted by three authors of this study—RA, MV, and GD—all of whom are experts in health psychology. These notifications gave participants feedback on their standings within the HAPA.

Participants in the “HAPA-NI group” received two daily notifications (delivered at 10 a.m. and 4 p.m.) that emphasized HAPA variables (as described in the Supplementary material), but they did not interact with the app. For example, one of the notifications focused on Action Self-Efficacy, stating, “Feeling capable of achieving your goal through good planning and effective strategies is an excellent start.” The notifications for both the HAPA-I and HAPA-NI groups were identical in structure, word count, and graphic format. The only difference was the level of content customization: it was generic for the HAPA-NI group and shaped based on participants’ responses for the HAPA-I group.

The “No-Communication Group” did not receive HAPA-based notifications during the trial period.

**Figure 2:**
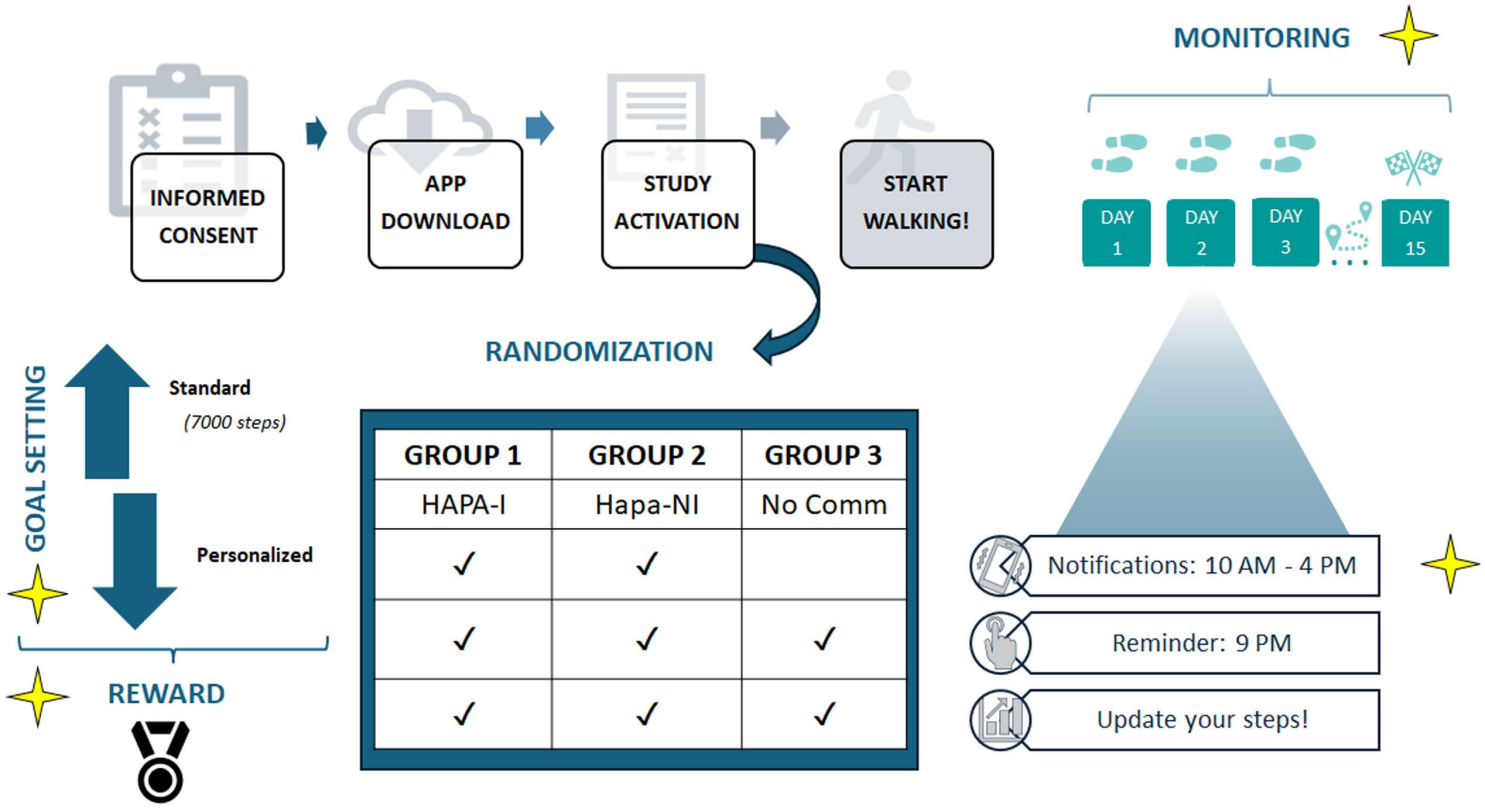
Trial procedure.

All participants, regardless of their experimental condition, received a daily reminder at 9 p.m., in the form of a notification, to log the number of steps they took each day in a dedicated section of the app for walking self-monitoring. As participants entered their step counts, a graph gradually filled, illustrating the temporal trend of their behavior relative to the set goal. Additionally, when participants achieved their goal for the day, they were awarded a medal and received the following motivational message: “Congratulations! Today went great; keep it up!” Figure 2 illustrates the procedure.

### 2.3 Assessment of walking behavior

The International Physical Activity Questionnaire (IPAQ; Craig et al., 2003; Mannocci et al., 2010) was used in the online recruitment questionnaire to identify inactive volunteers. The IPAQ consists of seven items that evaluate sedentary behavior and three specific types of physical activity: walking, moderate-intensity, and vigorous-intensity activity. These items measure the frequency (days per week) and duration (minutes per day) of each activity. The structure of the IPAQ allows for separate scores for each activity type, as well as a combined total score that indicates the overall activity level in terms of median MET (Metabolic Equivalent of Task) minutes per week. These scores are calculated by weighing the amount of physical activity against the energy requirement, defined in METs, which are multiples of resting metabolism. For this study, a cut-off of 3,000 MET-minutes/week was set to classify respondents as inactive or minimally active. Volunteers who exceeded this threshold were excluded from participation.

Moreover, to ensure that only volunteers who were inactive in relation to the specific type of physical activity proposed by the study were included, the recruitment questionnaire explicitly asked whether participants usually took at least 7,000 steps per day. Those who answered “yes” were excluded from participation.

Participants tracked their walking behavior daily. Each evening, the app prompted them to enter the number of steps they had taken, as reported by their smartwatches. To evaluate the effectiveness of the app-based intervention in increasing participants’ daily step counts, the average number of steps achieved in the first 3 days of the intervention was considered as a “starting point” for statistical analyses. This decision was based on the understanding that asking participants to report the average number of steps taken before the intervention (as a baseline) would likely yield unreliable measurements, as not everyone regularly tracks their daily steps. Instead, considering the average number of steps in the first 3 days as a starting point was deemed more effective to measure the change over time since the start of the intervention, as it provides a stable and reliable reference for step count while accounting for individual activity patterns and daily fluctuations. We then analyzed the variation in daily step counts compared to this starting point. This measure served as the primary outcome for assessing the effectiveness of the intervention delivered through MyPocketHealth.

Since the intervention was conducted in a real-world setting, we introduced a measure to assess external factors that may have influenced participants’ walking habits. In the T1 questionnaire, we included the following question: “Over the past 2 weeks, we asked you to try to increase your daily step count. Aside from this activity, have any changes in your routine affected your daily steps? For example, were you on vacation, unwell, or did other events lead you to walk more or less?” Participants could respond with one of the following options: “No, there were no changes,” “Yes, there were circumstances that caused me to walk more,” or “Yes, there were circumstances that caused me to walk less.”

### 2.4 Engagement with the intervention and usability of the application

Participants’ engagement with the intervention was assessed in the final online questionnaire (T1) by asking participants to rate their overall experience using a 7-point Likert scale. This rating was based on three pairs of adjectives in a semantic differential scale adapted from Maggino and Mola (2007). The pairs of adjectives included: “Difficult” vs. “Easy,” “Useless” vs. “Useful,” and “Boring” vs. “Stimulating.” A higher score indicated a more positive evaluation. The mean score was calculated, demonstrating good internal consistency (McDonald’s *ω* = 0.82).

The app’s usability was evaluated in the final online questionnaire (T1) using the System Usability Scale (SUS), a tool developed by Brooke (1996). This scale measures the perceived usability of various devices and systems. It consists of 10 items, each rated on a 5-point Likert scale (1 = completely disagree and 5 = completely agree). The scoring for each item ranges from 0 to 4. To calculate the total score, raw scores are recoded, summed up, and then multiplied by 2.5. Higher total scores indicate a greater perceived usability. Over time, adaptations to the scoring procedures have been proposed, including a letter-grade translation of the scores based on a normal distribution of mean scores. The grading scale is as follows: A = excellent usability (scores > 80.3), B = good usability (80.3–68), C = fair usability (68), D = acceptable usability (67–51), and F = poor usability (scores < 51) (Lewis, 2018).

### 2.5 Data analysis

Analyses were performed using Jamovi, version 2.6.44 (https://www.jamovi.org/, accessed on April 23, 2025). All statistical tests were two-tailed, and a *p* ≤ 0.05 was considered statistically significant.

We calculated descriptive statistics on the sample’s sociodemographic and behavioral characteristics. We reported mean and standard deviation (SD) for continuous variables and percentages for categorical variables. Normality of the data was assessed using skewness and kurtosis indices, with recommended ranges of ±2 and ±7, respectively (Hair et al., 2010). McDonald’s *ω* was calculated to estimate the internal consistency of the engagement scale (McDonald, 2013).

Attrition is expected in longitudinal studies. Accordingly, the Mann–Whitney U and Chi-square tests were used to identify possible differences between participants who completed the study and those who did not complete the T1 online questionnaire.

The objectives of this study were addressed through the following analyses:

- A linear mixed model (LMM) with restricted maximum likelihood (REML) estimation, as implemented in Jamovi (Gallucci, 2019), using the Satterthwaite method for degrees of freedom, was conducted to evaluate the effectiveness of the intervention delivered through the app. The analysis specifically aimed to assess whether walking behavior improved over the course of the intervention (first hypothesis) and whether the type of communication—HAPA-I, HAPA-NI, or No comm—moderated this effect (second hypothesis). Additionally, the model controlled for potential influences from sociodemographic factors and external variables that might have affected participants’ walking habits. The variation in daily step counts compared to the starting point served as the dependent variable. Independent variables included sociodemographic characteristics such as gender (two levels: female and male) and age (as a covariate), external factors affecting walking habits (three levels: no changes, walked more, walked less), time (as a covariate), group (three levels: HAPA-I, HAPA-NI, and no communication), and the interaction between time and group. A random slope was included to account for interindividual differences.
- Two one-way ANOVAs were conducted, using participant engagement with the intervention and the application’s usability as the dependent variables (third hypothesis). The independent variable in each analysis was the group variable (three levels).

## 3 Results

### 3.1 Preliminary analyses

All continuous variables considered in this study had distributions that approximated normality.

Volunteers who dropped out of the study did not differ significantly from the participants in their sociodemographic characteristics, namely age, gender, education, or working status (*p* > 0.05). They did not differ in the walking behavior declared in the recruitment questionnaire. Participants differed in group allocation [*χ*^2^(2) = 23.61, *p* < 0.001; Cramer’s V = 0.29]. The dropout rate was higher in the HAPA-NI group (45%) compared to the HAPA-I (16%) and No communication (21%) groups.

### 3.2 Description of walking behavior

On average, participants took 6,041 steps (SD = 2,777) during the first 3 days of the intervention. Walking increased to an average of 6,257 steps (SD = 3,150) during the intermediate period, which spanned from the fourth to the ninth day. In the final period of the intervention, between the tenth and fifteenth days, participants averaged 6,436 steps (SD = 2,861).

Nearly half of the participants (48%) set a goal of fewer than 7,000 steps, while 42% aimed for exactly 7,000 steps. Only a small portion of the sample (10%) set a goal of more than 7,000 steps.

The distribution of medals—awarded for the number of days participants met their step goals—indicates that 25% of participants earned five or fewer medals, while 50% (the median) earned nine medals. Additionally, 75% of participants earned 13 or fewer medals. This distribution suggests that half of the participants earned between 5 and 13 medals, with a median of 9. The relatively equal distances between the percentiles indicate a roughly symmetrical distribution, with no significant imbalance between the lower and upper tails.

### 3.3 Effectiveness of the intervention

**Figure 3:**
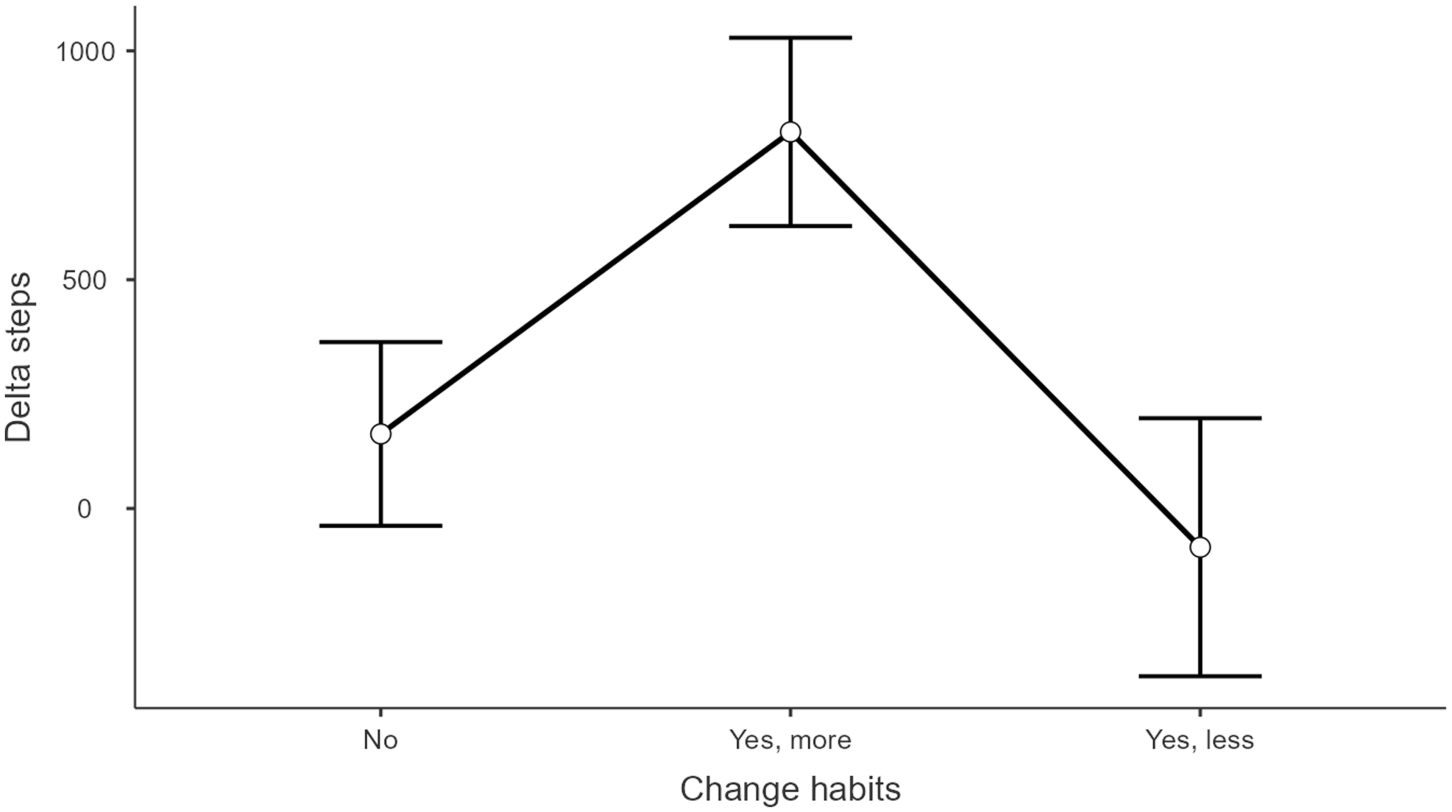
Marginal means of the variation in daily step counts compared to the starting point as a function of the presence of external factors that influenced participants to walk more or less during the intervention. The bars represent standard errors.

**Figure 4:**
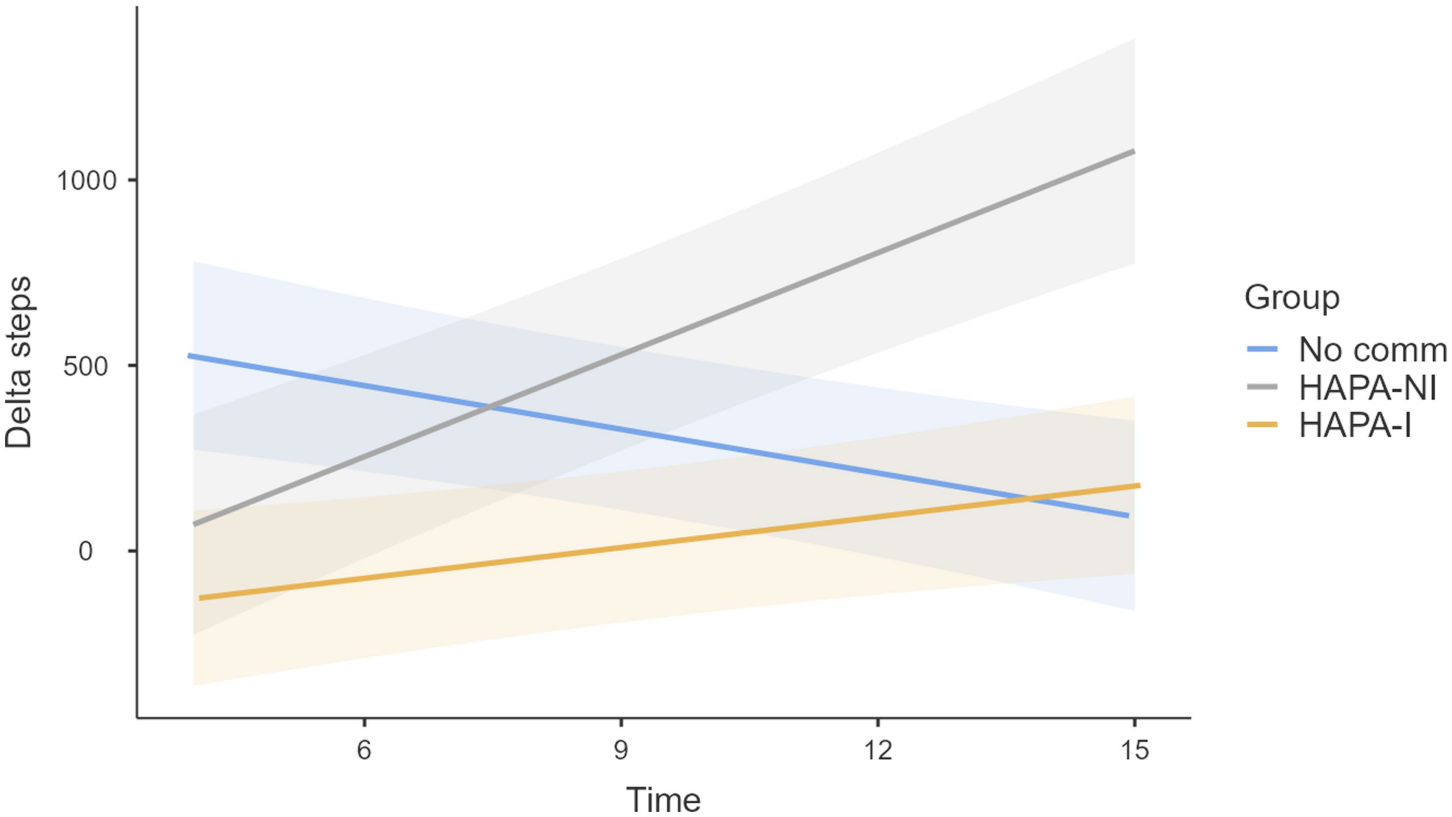
Marginal means of the variation in daily step counts compared to the starting point as a function of time and group. The bands represent standard errors.

The LMM successfully converged and explained 35% of the variance (*R*_c_^2^ = 0.35). The fixed effects explained 3% of the variance alone (*R*_m_^2^ = 0.03). Fixed effects are summarized in Table 2. The factor “change habits” was significant [*F*(2, 201) = 4.44, *p* < 0.05]. Participants who experienced circumstances outside the intervention that encouraged them to walk more during the intervention showed a greater increase in the number of steps taken (mean = 823; SE = 206) compared to those who did not experience significant external circumstances (mean = 163; SE = 201) or those who experienced circumstances that led them to walk less (mean = −85; SE = 282), as illustrated in Figure 3. The “group × time” interaction [*F*(2, 2,172) = 6.52, *p* < 0.005] suggested that, in the HAPA-I and HAPA-NI groups, an increase in the variation of the mean number of daily steps was observed over time, while the No comm group showed a decline (Table 2 and Figure 4). The likelihood ratio test (LRT) indicated that the inclusion of a random intercept to account for interindividual differences significantly improved the model (LRT = 538.65; *p* < 0.001). The ICC was considerable (0.34), indicating that 34% of the total variance was attributable to interindividual differences, justifying the inclusion of a random intercept.

**Table 2:** Linear mixed model of the variation in daily steps count: parameter estimates (fixed coefficients).

				95% Confidence Intervals				
Names	Effect	*β*	SE	Lower 95% CI	Upper 95% CI	df	*t*	*p*
(Intercept)	(Intercept)	−0.01	0.05	−0.11	0.09	200.64	−0.22	0.828
Gender	Man - Woman	0.08	0.10	−0.11	0.27	199.26	0.81	0.421
Age	Age	0.04	0.04	−0.05	0.12	199.88	0.87	0.385
Change habits	Yes, more - No	**0.23**	**0.10**	**0.04**	**0.42**	**199.78**	**2.34**	**0.020**
	Yes, less - No	−0.09	0.12	−0.31	0.14	201.19	−0.75	0.456
	Yes, more - Yes, less	**0.32**	**0.12**	**0.09**	**0.55**	**201.29**	**2.68**	**0.008**
Group	HAPA-NI - No comm	0.09	0.11	−0.13	0.31	201.10	0.82	0.411
	HAPA-I - No comm	−0.10	0.10	−0.30	0.10	200.10	−0.99	0.325
	HAPA-NI - HAPA-I	0.19	0.11	−0.02	0.41	200.86	1.74	0.083
Time	Time	0.03	0.02	−0.00	0.07	2172.16	1.87	0.062
Group * Time	(HAPA-NI - No comm) * Time	**0.16**	**0.04**	**0.07**	**0.24**	**2172.54**	**3.59**	**<0.001**
	(HAPA-I - No comm) * Time	**0.08**	**0.04**	**0.00**	**0.16**	**2170.93**	**2.05**	**0.040**
	(HAPA-NI - HAPA-I) * Time	0.08	0.04	−0.01	0.16	2172.78	1.80	0.072

Significant effects are highlighted in bold. *β*, standardized estimates.

### 3.4 Engagement with the intervention and usability of the application

Overall, participants expressed good engagement with the intervention, achieving a mean score of 5.65 (SD = 1.36) on a scale from 1 to 7. The perceived usability of the application was also high, with a mean score of 79.56 (SD = 17.17), indicating good usability. The ANOVA results showed no significant differences between the experimental groups in terms of engagement or app usability (*p* > 0.05).

## 4 Discussion

Advances in theory-driven behavior change techniques and interventions targeting health-related behaviors are crucial for developing effective primary prevention strategies (Michie et al., 2011). Among modifiable health risk factors, physical inactivity has garnered considerable attention, leading to the development of innovative behavior change interventions delivered through mobile Health (mHealth) technologies (Davis et al., 2020; Singh et al., 2024). These approaches have produced encouraging results. However, specific issues emerged regarding the actual effectiveness of digital tools in sustaining behavior change (Rodríguez-González et al., 2023; Maes et al., 2024; Pomkai et al., 2024). This study is situated within the context of this research and builds upon a broader line of investigation (Adorni et al., 2025).

We employed a prospective, three-arm, parallel-group, randomized study design to evaluate the effectiveness of a multicomponent, theory-based mHealth intervention that includes goal setting, goal review, self-monitoring, and rewards for successful behavior. Additionally, we specifically examined the moderating effect of two types of digital communication modalities differing in interactivity.

A notable first finding was higher participant attrition in the HAPA-NI group than in the other two experimental conditions. This result suggests that receiving two daily notifications that do not require any interaction with the app (HAPA-NI) may be more burdensome than either receiving no notifications (No Comm) or receiving interactive prompts that require user responses (HAPA-I). Research indicates that push notifications do not always enhance user retention; in some cases, they may even lead to shorter app usage or dropout, particularly when notifications are too frequent or lack relevance or interactive content. For example, in their systematic review, Jakob et al. (2022) highlighted that one factor positively influencing adherence is the design of reminders in the form of individualized push notifications. Consistently, Woodward et al. (2021) conducted a survey on notification design in commercial applications and found that high daily notification frequency can actually decrease retention. They also noted that using non-personalized content can lead to reduced user engagement. This evidence emphasizes the importance of both frequency and personalization in notifications to prevent potential dropouts.

Most participants set a goal of fewer than 7,000 steps (48%) or exactly 7,000 steps (42%). Overall, half of the participants maintained their goals for 9 out of the 15 days outlined by the intervention. On the one hand, these results are encouraging, especially given that the sample consisted of physically inactive individuals who were not accustomed to walking in their daily lives. On the other hand, given that participants chose to download the app and engage in the intervention, the relatively modest change observed confirmed that improving physical activity is complex, as noted in prior research (World Health Organization, 2024a).

Upon closer examination of the results, the study’s first hypothesis was not fully supported. The increase in the number of steps observed did not achieve statistical significance within the mixed linear model; it only indicated a trend. However, the second hypothesis was confirmed: the experimental condition significantly moderated the change in the average number of steps during the intervention compared to the starting point. This effect was statistically significant. In the No Comm group, a significant increase in the average number of daily steps was observed compared to the starting point during the initial days of the intervention; however, this effect progressively attenuated over time. By contrast, participants in the two groups receiving HAPA-based notifications exhibited only modest initial changes relative to the starting point, which tended to increase in strength as the intervention progressed. Taken together, these findings suggest that receiving notifications reinforcing the HAPA constructs may have supported participants’ sustained commitment to their behavioral goals over time. This result aligns with the literature, which emphasizes the importance of building interventions on a solid theoretical foundation (Michie et al., 2011). It shows that HAPA-based interventions are particularly relevant for the maintenance phase of behavior (Wunsch et al., 2024).

We hypothesized that exposure to the HAPA-I condition would be more effective than exposure to the HAPA-NI condition; however, the results did not support this expectation. Overall, participants in the HAPA-I condition were more engaged in the study and had higher retention rates. However, the HAPA-NI condition appeared to influence participants’ behavior more positively than the HAPA-I condition (see Figure 4). The interpretation of this evidence may be in the higher dropout rate in the HAPA-NI group: it is plausible that those who remained engaged were more intrinsically motivated to achieve their goals over time. This observation suggests a potential attrition bias among participants in the HAPA-NI group who completed the study (Hausman and Wise, 1979).

Another important observation is that the moderating effect of experimental group assignment on walking behavior was statistically significant, even after accounting for potential confounding factors related to external circumstances. Accounting for external circumstances such as vacations or illness is particularly relevant in real-world physical activity interventions, where behavior is embedded in participants’ everyday lives and experimental control is necessarily limited (Glasgow et al., 2003). Previous research has shown that walking behavior is highly sensitive to situational and temporal factors (Tudor-Locke et al., 2011). This evidence highlights the importance of modeling situational events in longitudinal studies to distinguish between “context” effects and “intervention” effects (Michie et al., 2018). The fact that the moderating effect of experimental group assignment remained statistically significant after controlling for these influences strengthens the interpretation that the observed trajectory reflects an intervention-related effect rather than contextual fluctuations.

The analysis revealed no significant effects of gender or age on changes in step count over time. This result is consistent with findings from our previous study (Adorni et al., 2025) and may be explained by several factors. First, it is plausible that all participants, irrespective of gender or age, were equally motivated to adhere to the intervention once enrolled. Moreover, it is worth noting that the study sample was relatively narrow in age (18–55 years). This methodological choice aimed to exclude older adults who might have experienced difficulties due to limited familiarity with mobile technologies, as evidenced by previous literature (Maes et al., 2024). Regarding gender, women constituted the majority of the sample (69%). While this imbalance may have reduced the ability to detect gender-related statistical differences, it also mirrors the higher levels of engagement typically observed among women in mHealth research, as reported in previous studies (Zhang et al., 2022; Bonn et al., 2024).

Regarding the third hypothesis of the study, the findings indicate that the app demonstrated good usability and that participants reported a positive overall experience with the intervention. They perceived the mHealth intervention as relatively easy, helpful, and stimulating, suggesting that users accepted it. High usability is a crucial factor in the effectiveness of digital health interventions, while poor usability can significantly affect user engagement, adherence, and the long-term effectiveness of the intervention (Eaton et al., 2024). In this regard, the present findings are particularly relevant, as they indicate that participants perceived the app as easy to use, reducing the cognitive and technical barriers that often limit sustained interaction with digital tools (Palos-Sanchez et al., 2021; Mahajan et al., 2025). Moreover, the perceived usefulness of the intervention suggests that users recognized its relevance and value in supporting their walking behavior, which may enhance motivation and increase the likelihood of continued use (Buawangpong et al., 2025). Additionally, the positive perception of the intervention as stimulating underscores the importance of user experience in fostering engagement with health-related technologies. An intervention that is perceived not only as functional but also as motivating may foster greater involvement and facilitate the adoption of the targeted behavior (Bergevi et al., 2022). Notably, the specific experimental condition did not influence these evaluations. This result may be explained by a shared core structure across experimental groups, as all participants received the same multicomponent intervention, including four BCTs. These common elements may have had a dominant influence on participants’ perceptions, reducing the likelihood that differences between experimental conditions would emerge in terms of usability and overall appreciation. In addition, measures of perceived usability and intervention judgment may be more sensitive to general features of the user experience rather than to differences in specific BCT implementations, thereby capturing a more global evaluation of the intervention (Wang et al., 2025). It is important to note that a significant portion of volunteers (28%) dropped out of the study and did not complete the final questionnaire, which included questions related to engagement and usability. This evidence suggests being cautious about the above considerations, as they could be affected by an attrition bias (Hausman and Wise, 1979) within the sample.

This study has some limitations. First, we used self-report methods to assess most variables (e.g., eligibility and HAPA interactive notifications). While self-report measures can be influenced by social desirability bias (Chukwuemeka et al., 2025), they are the most viable option for a large-scale app-based intervention like the one described in this study. This methodology is practical, cost-effective, and effectively captures individuals’ subjective experiences (Prince et al., 2008). Notably, daily step counts provided a more objective indicator of walking behavior than self-report questionnaires, advancing a standardized, real-time evaluation of the primary outcome. Second, the samples in this study were predominantly female, consistent with previous research in this field (Zhang et al., 2022; Bonn et al., 2024). Gender differences should be carefully considered, as men and women may exhibit distinct behavioral patterns and psychological characteristics that could influence intervention outcomes. Future studies should aim to recruit more gender-balanced samples to explicitly control for gender-related variables (Zhang et al., 2022), thereby enhancing the generalizability of the findings and ensuring that the observed effects more accurately reflect the intervention’s impact on the general population. Third, the lack of long-term follow-up limits the ability to determine whether the observed effects were maintained over time. Physical activity behaviors often fluctuate (Tudor-Locke et al., 2011), and sustained behavior change may require prolonged exposure or reinforcement. Future research should include follow-up assessments to examine the stability and long-term effectiveness of digital walking interventions (Zhang et al., 2022).

Despite its limitations, this study had several strengths. First, it addressed the crucial challenge of combating insufficient physical activity and sedentary behavior, both of which are essential for NCD prevention. The intervention promoted walking behavior among individuals, as recommended by Wunsch et al. (2024), by recruiting participants through a panel provider. This approach ensured that participants were neither personally known to nor in direct contact with the researchers and, together with the randomized design, strengthened the study’s methodological rigor and minimized potential sources of bias. Furthermore, external variables (e.g., personal routines, changes in habits, heavy rain, and unexpected commitments) were accounted for in the studied variables and the notifications’ content, as relevant factors affecting participants’ physical activity and study outcomes, consistent with the Ontology of Behavior Change Interventions model (Michie et al., 2018). Notifications were also grounded in the original definitions of the HAPA’s constructs, embedding them within a precise psychological theoretical framework. In line with Wang et al. (2025), who suggested using three or more BCTs simultaneously to maximize their effectiveness, four BCTs were applied. Moreover, following Zhang et al. (2022)’s recommendation, these BCTs were clearly specified and classified according to the CALO-RE taxonomy (2011). Finally, the requirement that participants possess a wearable device (i.e., a smartwatch) capable of tracking step count was included as an eligibility criterion. This choice ensured greater accuracy in step measurement while also guaranteeing that participants were already familiar with the use of such devices, thereby enhancing the reliability of the collected data.

In summary, the present study contributes to the growing body of research on theory-driven mHealth interventions for promoting physical activity by highlighting the role of digital communication modalities in shaping behavioral trajectories over time. Using a randomized, three-arm design, results demonstrated that notifications grounded in HAPA constructs can differentially influence the maintenance of walking behavior, supporting sustained engagement rather than the initial, short-lived increase observed in the absence of communication. These findings reinforce the importance of theoretically informed intervention components. Notably, the results show that the effectiveness of the intervention on walking behavior was detectable even after accounting for real-world contextual factors, underscoring the ecological validity of the findings. At the same time, the intervention was perceived as usable, helpful, and stimulating across all experimental conditions.

Taken together, these findings suggest that while core intervention components may primarily drive usability and overall user experience, variations in communication modality can play a critical role in sustaining behavior change over time. This study underscores the need to carefully balance frequency, interactivity, and theoretical relevance when designing digital notifications to maximize engagement and foster effectiveness.

## Data Availability

The raw data supporting the conclusions of this article will be made available by the authors, without undue reservation.

## Footnotes

1
https://www.bilendi.it
